# Real-Time Determination of the Cell-Cycle Position of Individual Cells within Live Tumors Using FUCCI Cell-Cycle Imaging

**DOI:** 10.3390/cells7100168

**Published:** 2018-10-14

**Authors:** Shuya Yano, Robert M. Hoffman

**Affiliations:** 1 AntiCancer, Inc., 7917 Ostrow street, San Diego, CA 92111, USA; shuyayano@okayama-u.ac.jp; 2 Department of Surgery, University of California San Diego, La Jolla, CA 92111, USA; 3 Department of Gastroenterological Surgery, Okayama University Graduate School of Medicine, Dentistry and Pharmaceutical Sciences, Okayama 700-0016, Japan

**Keywords:** cell cycle, fluorescent proteins, FUCCI, imaging

## Abstract

Most cytotoxic agents have limited efficacy for solid cancers. Cell-cycle phase analysis at the single-cell level in solid tumors has shown that the majority of cancer cells in tumors is not cycling and is therefore resistant to cytotoxic chemotherapy. Intravital cell-cycle imaging within tumors demonstrated the cell-cycle position and distribution of cancer cells within a tumor, and cell-cycle dynamics during chemotherapy. Understanding cell-cycle dynamics within tumors should provide important insights into novel treatment strategies.

## 1. Introduction

### 1.1. The Development of Intravital “Orthotopic” Imaging and a Fluorescent Cell Cycle Probe

Intravital fluorescence imaging enables visualization of live cancer cell dynamics including proliferation, invasion, and metastasis [1,2,3,4]. Our laboratory first demonstrated fluorescence imaging of cancer growth and metastasis using green fluorescent protein (GFP) [5,6,7], including lung cancer [8], pancreatic cancer [9], melanoma [10], ovarian cancer [11], and colon cancer [12]. Intravital real-time imaging, as well as whole-body imaging, was demonstrated [13,14,15]. Tsien et al. made various GFP mutants [16], which are brighter and show different colors, such as cyan, blue, and yellow. Multicolor fluorescent proteins enable subcellular imaging in vitro and in vivo [17,18,19,20,21,22,23]. Fluorescent proteins of different colors can monitor gene functions and cell fate, e.g., Yang et al. [24] and Amoh et al. [25] demonstrated that nestin-driven GFP transgenic mice visualized tumor blood vessels. Schepers et al. demonstrated that tracing LGR5 intestinal stem cells visualized the fate of stem cells for intestinal adenomas and early-stage cancer in GFP-LGR5 transgenic mice with multiple colors [26]. Livet et al. demonstrated “brainbow” labeling of individual cells in the analysis of neuron circuitry [27]. Imaging instruments for intra-vital imaging include multi-photon laser microscopes for visualizing live single cells in tumors [28,29,30]. 

Our laboratory has developed in vivo intravital imaging techniques of tumors growing orthotopically in the brain, liver, other organs of mice, and circulating cancer cells in blood vessels and lymphatic ducts during metastasis [1,2,13,17,20,21]. In vivo intravital imaging also demonstrated the difference in behavior of cancer cells at the orthotopic and subcutaneous sites in real-time [31,32,33,34,35]. 

In this review, we focus on intravital imaging with the fluorescence ubiquitination-based cell cycle indicator (FUCCI) imaging of individual cells in tumors. Intravital FUCCI-imaging provides a new paradigm of cell-cycle-based treatment of solid cancers.

### 1.2. FUCCI (Fluorescence Ubiquitination Cell Cycle Indicator) Repoters

**Figure 1:**
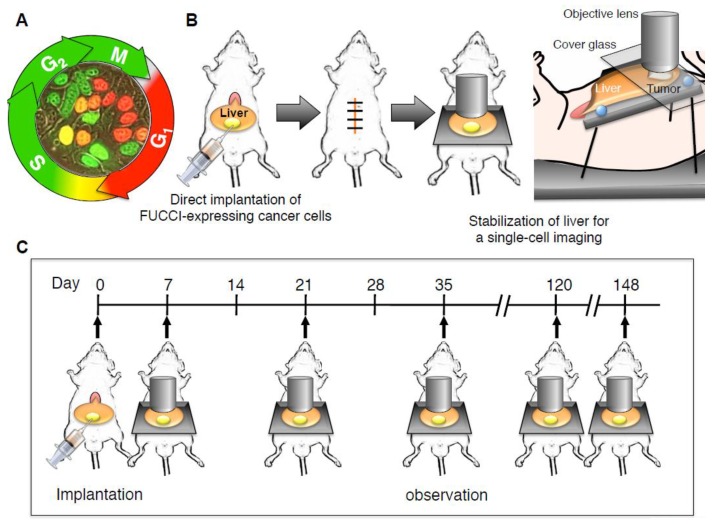
An abdominal skin-flap window for longitudinal intravital imaging of fluorescence ubiquitination-based cell cycle indicator (FUCCI)-expressing cancer cells in the liver of a live mouse. (**A**) FUCCI-expressing MKN45 gastric cancer cells in G_0_/G_1_, S, or G_2_/M phases are red, yellow, or green, respectively. (**B**) The schematic diagram shows the method of longitudinal intravital confocal laser scanning microscopy (CLSM) imaging of FUCCI-expressing gastric-cancer cells growing in the liver using a skin-flap window. All animal procedures were performed under anesthesia using s.c. administration of a ketamine mixture (10 μl ketamine HCl, 7.6 μl xylazine, 2.4 μL acepromazine maleate, and 10 μL PBS). FUCCI-expressing MKN45 cells were harvested by brief trypsinization. Single-cell suspensions were prepared at a final concentration of 2 × 10^5^ cells/5 μl Matrigel (BD). After laparotomy, FUCCI-expressing cancer cells were subserosally injected directly into the left lobe of the liver using a 31 gauge needle. After cancer-cell implantation, the abdominal wall of the mice was closed with 6-0 sutures. Mice were anesthetized as described above and placed on a custom-designed imaging box. The liver was exteriorized and placed on a Styrofoam box, and a cover glass was gently placed on the liver, which inhibited vibration caused by heartbeat and respiratory movement. CLSM imaging was performed using the FV1000 confocal laser microscope (Olympus Corp, Tokyo, Japan) with two-laser diodes (473 nm and 559 nm). A 4 × (0.20 numerical aperture immersion) objective lens and 20 × (0.95 numerical aperture immersion) objective lens (Olympus) were used. 800 × 800 pixels and 1.0 μm z steps were scanned, which took 1–2 s per section. Scanning and image acquisition were controlled by Fluoview software (Olympus). (**C**) The schematic diagram shows the method of longitudinal intravital CLSM imaging of FUCCI-expressing gastric cancer cells growing in the liver using an abdominal skin-flap window. The abdominal skin-flap window method enables ten laparotomies during 150 days.

Sakaue-Sawano et al. [36] have reported that the cell-cycle phase in viable cells can be identified using FUCCI. Red nuclei in FUCCI-expressing cells (FUCCI-red) indicate the quiescent G_0_/G_1_ phase [37], green nuclei in FUCCI-expressing cells (FUCCI-green) indicate the proliferating late-S/G_2_/M phase, and yellow nuclei in FUCCI-expressing cells (FUCCI-yellow) indicate the early S phase (Figure 1A). Intravital FUCCI imaging enabled visualization of cell-cycle dynamics of individual cancer cells within tumors. Moreover, FUCCI imaging also visualized cell-cycle dynamics within tumors during chemotherapy. Sakaue-Sawano et al. also demonstrated that the FUCCI 2 that emits red (mCherry) and green (mVenous) fluorescence and provides better color contrast than original FUCCI [38]. Bajar et al. modified the original FUCCI in order to visualize four cell-cycle phases [39]. Furthermore, Sakaue-Sawano et al. [40] recently developed two new FUCCI; FUCCI (CA); and FUCCI (SCA). FUCCI (CA) produced a sharp triple-color distinct separation of G_1_, S, and G_2_, while FUCCI (SCA) permitted a two-color readout of G_1_ and S/G_2_ phases. Oki et al. [41] modified FUCCI, which enabled the distinction between G_0_ and G_1_. A bright fluorescence signal is the most important for intravital imaging in vivo. Moreover, these fluorescent proteins should not be easily bleached. The original FUCCI for intravital real-time in vivo imaging of cancer cells at the single cell level met these criteria.

## 2. Longitudinal Intravital Imaging of an Orthotopic Metastatic Liver Tumor Model with FUCCI

### 2.1. Skin-Window System

The stabilization of a target organ is one of the most indispensable steps for single-cell live imaging with a confocal laser microscope (Olympus Corp, Tokyo, Japan). Cross-sections are needed for single-cell imaging. Chittajallu et al. [42] demonstrated that a dorsal skin-fold-chamber enabled visualization of drug response of FUCCI-expressing HT1080 soft-tissue sarcoma. Ritsma et al. [43,44] also demonstrated that an abdominal window using a coverslip enabled visualization of the biological behavior of Colo26 mouse colon cancer cells in the liver of a mouse. Both window methods are useful and convenient for intravital single-cell imaging. However, a coverslip window limits the area for imaging [45], and the glass pressed on the cancer cells may affect their behavior in vivo. 

### 2.2. Minimal Organ-Stabilization System Using Styrofoam and Pins

Therefore, Yano et al. [46] developed a convenient, minimally-invasive organ stabilization system using a styrofoam board, tape, pins, and a cover glass over the liver of a mouse (Figure 1B). This system enabled laser-scanning microscopy imaging of cancer cells in the liver of the live mouse without vibration caused by heartbeat and respiratory movement of a mouse under anesthesia. This system also enabled tracing the same location and the same cancer cells at the single-cell level in a live mouse, even with repeat laparotomies (Figure 1C). 

### 2.3. Cell-Cycle Distribution within a Tumor 

**Figure 2:**
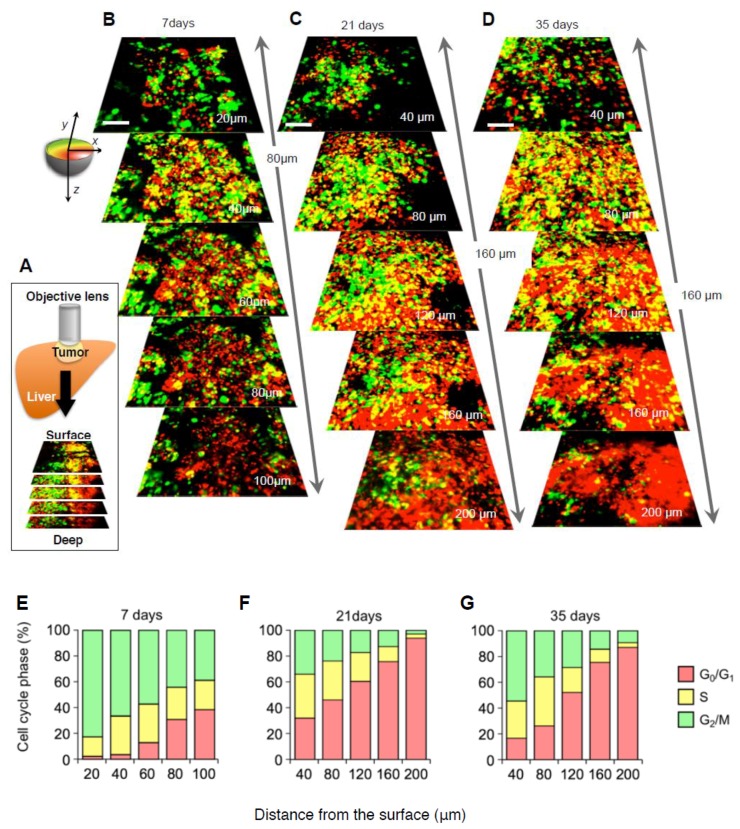
Intravital cell cycle imaging in FUCCI-expressing tumors shows the cell-cycle phase distribution of cancer cells at the tumor surface and center. High-resolution images (tile scan) were obtained with a 20 × (0.95 numerical aperture immersion) objective lens. To monitor the cell-cycle distribution of cancer cells during tumor growth, three-dimensional images (z stacks) of the same tumor at day 7, 28, and 90 post-implantation were used. (**A**) The schematic diagram shows the method of longitudinal intravital CLSM imaging of FUCCI-expressing MKN45 gastric-cancer cells growing in the liver using a skin-flap window. (**B**–**D**) FUCCI-expressing MKN45 cells were implanted directly in the liver of nude mice and imaged at 7 days (**B**), 21 days (**C**), and 35 days (**D**). (**E**–**G**) Histograms show the distribution of FUCCI-expressing cells at different distances from the surface. The number of cells in each cell-cycle phase was assessed by counting the number of cells of each color at the indicated time points and depth. The percentage of cells in the G_2_/M, S, and G_0_/G_1_ phases of the cell cycle are shown. Scale bars represent 100 μm. Data are means ± SD. (Reproduced from [46] with the permission of Taylor and Francis).

Monitoring cell cycle dynamics during tumor growth is very important for improving our understanding of cancer. Yano et al. [46] demonstrated intravital real-time monitoring of orthotopic FUCCI-expressing tumors in the liver of live mice during tumor growth (Figure 2A). Nascent tumors (7 days after inoculation) consisted of a majority of proliferating cancer cells (Figure 2B,E). Quiescent cancer cells increased during tumor growth (Figure 2C,F). In contrast, the vast majority of cells in an established tumor are quiescent (Figure 2D,G). In an established tumor, proliferating cancer cells exist only at the surface area (Figure 2C,D). Chittajallu *et al.* demonstrated intravital single-cell imaging of FUCCI-expressing HT1080 fibrosarcoma cells implanted in a dorsal skin-fold-chamber in nude mice. Chittajallu et al. [42] clearly visualized G_1_, late G_1_/early S, S/G_2_, and mitosis in vivo combining a nuclear-morphology reporter (histone H2B-CFP) and the FUCCI system. Chittajallu et al. [42] could image for only two weeks, since tumor growth was limited to the depth of the chamber.

### 2.4. Established Tumors Consist of a Vast Majority of Quiescent Cancer Cells

**Figure 3:**
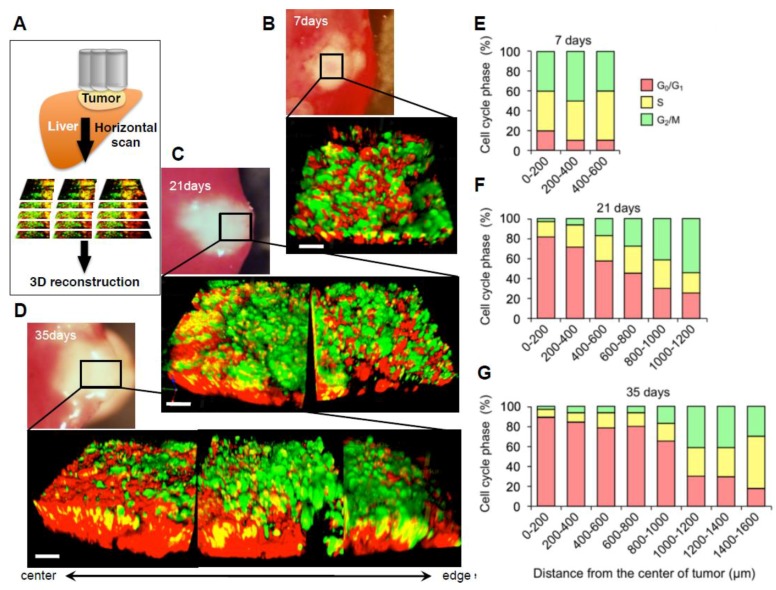
Three-dimensional image of FUCCI-expressing tumor reveals a vast majority of quiescent cancer cells. (**A**) Schematic diagram of in vivo CLSM imaging of different-sized tumors. Tumors were scanned from the center to the edge. 800 × 800 pixels and 1.0 μm z steps were scanned, which took 1–2 s per section, with 6–8 min per full 3D scan. The tracing data were imported to Velocity 6.0 version (Perkin Elmer), where all further analyses were performed, and then the scanned images were three-dimensionally reconstructed. (**B**–**D**) Representative 3D reconstruction images of a nascent tumor at 7 days after cancer-cell implantation (**B**), 21 days (**C**), and 35 days (**D**) after implantation. (**E**–**G**) Histograms show the distribution of FUCCI-expressing cells at different distances from the center. The number of cells in each cell-cycle phase was assessed by counting the number of cells of each color at the indicated time points. The percentage of cells in the G_2_/M, S, and G_0_/G_1_ phases of the cell cycle is shown. Scale bars represent 100 μm. (Reproduced from [46] with the permission of Taylor and Francis).

Solid tumors are well known to be heterogeneous, which makes it difficult to understand cancer biology [47,48]. Our abdominal skin-flap method enabled reconstruction of three-dimensional images (Figure 3A) [46]. Yano et al. [46] showed that a nascent tumor (7 days after inoculation) consisted of cells that were mostly (90%) in S/G_2_/M (Figure 3B,E). In contrast, a medium-sized established tumor (21 days after inoculation) had regions of both G_2_/M cells (65 to 30%) and G_0_/G_1_ cells (35 to 70%) (Figure 3C,F). Furthermore, a large-sized tumor (35 days after implantation) consisted of cells that were mostly (90%) in G_0_/G_1_ (Figure 3D,G). The surface of the tumor consisted of cells mostly (70 ~ 80%) in S/G_2_/M regardless of the time after implantation and tumor size, indicating the cancer cells near the tumor surface were mostly cycling and growing outward. These results indicate that most cancer cells in nascent tumors are cycling. As the tumor becomes larger, most cancer cells become quiescent. Chittajallu et al. [42] used FUCCI imaging of tumors and confirmed our results. 

## 3. Intravital Orthotopic FUCCI Imaging Reveals the Relationship between Cell Cycle Phase of Cancer Cells and the Juxtaposition of Tumor Blood Vessels

**Figure 4:**
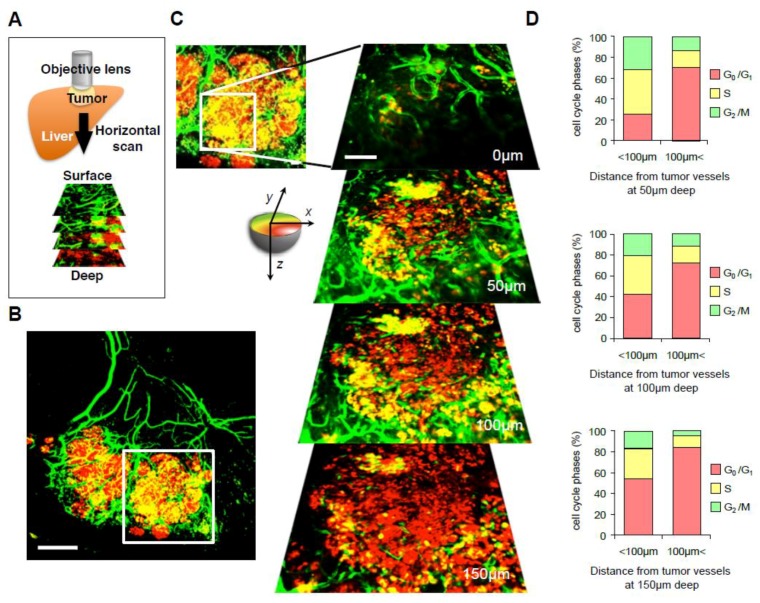
Imaging nascent tumor vessels and cancer cell-cycle phase. (**A**) The schematic diagram shows the method of repeated intravital CLSM imaging of FUCCI-expressing cells growing in nestin-driven green fluorescent protein (ND-GFP)-expressing transgenic nude mice in which tumor nascent vessels express GFP. (**B**) Representative whole image of orthotopic FUCCI-expressing tumor in ND-GFP-expressing nude mice. (**C**) Representative images of FUCCI-expressing cancer cells at various depths in a tumor in the liver at 28 days after implantation are shown. (**D**) Histogram shows the cell cycle phase distribution at different distances (xy-plane) from blood vessels. Scale bars represent 100 μm. (Reproduced from [46] with the permission of Taylor and Francis).

It is also important to investigate the relationship between cancer cells and tumor blood vessels [49]. Kienast et al. [50] demonstrated intravital single-cell imaging of multistep-brain metastasis of cancer cells using a combination of a multiphoton laser microscope and a cranial window. Kienast et al. [50] showed that cancer cells are initially arrested at a blood vessel branch, when they extravasted, and then grew at the perivascular position with angiogenesis. To investigate the cell-cycle position of cancer cells near and far from vessels, transgenic mice with nestin-promoter driving GFP (nestin-driven GFP [ND-GFP]) were used to label nascent blood vessels with GFP [24,25] (Figure 4A,B). Yano et al. [46,51] also reported that proliferating cancer cells exist only near tumor vessels or the tumor surface; in contrast, cancer cells far from vessels or in the center of tumors are quiescent (Figure 4C,D).

## 4. Intravital Orthotopic FUCCI Imaging Reveals that Quiescent Cancer Cells are Resistant to Conventional Chemotherapy

**Figure 5:**
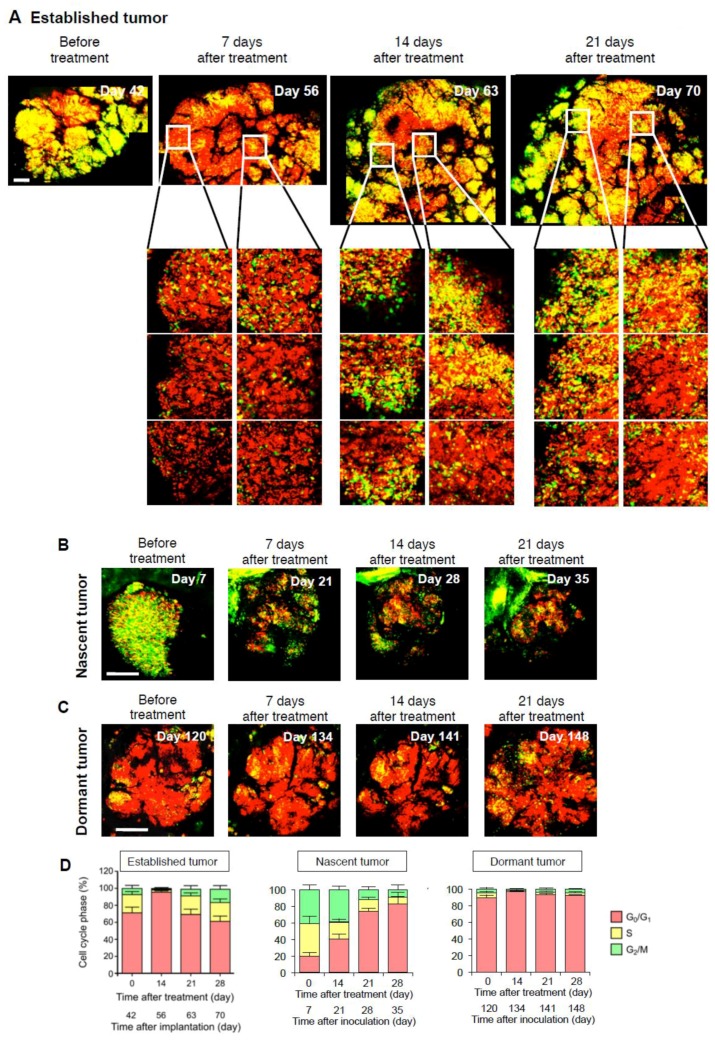
Response to cytotoxic chemotherapy of cells in various phases of the cell cycle within tumors. Representative images of a FUCCI-expressing tumor in the liver before and after cisplatinum (CDDP) treatment. (**A**) Representative images of an established FUCCI-expressing tumor in the liver before and after CDDP treatment. (**B**) Representative images of a nascent FUCCI-expressing tumor in the liver before and after CDDP treatment. (**C**) Representative images of a dormant FUCCI-expressing tumor in the liver before and after CDDP treatment. (**D**) Histogram shows the cell cycle phase distribution of cancer cells in the tumor at the indicated time points. Data are means ± SD (each group for *n* = 5). Scale bars represent 500 μm. (Reproduced from [46] with the permission of Taylor and Francis).

Resistance to conventional chemotherapy is an important clinical problem that results in tumor recurrence and poor prognosis in cancer patients. Most currently-used anticancer agents are effective only on cycling cancer cells [52] and have no effect on quiescent/dormant cancer cells [53,54,55,56,57,58]. To overcome chemoresistance, intravital imaging was performed [59,60,61,62,63]. The cell-cycle phase determines the cancer-cells response to chemotherapy [64,65,66,67,68,69,70]. Yano et al. [46] demonstrated by FUCCI imaging that currently-used cytotoxic anticancer agents are ineffective for solid tumors, since they comprise mostly non-cycling, quiescent cancer cells (Figure 5). Chittajallu et al. [42] confirmed our results using intravital single-cell level FUCCI imaging in nude mice. Intravital FUCCI imaging determined that the cell cycle phase distribution of cancer cells in vivo differed from 2D in vitro cell culture.

## 5. Intravital Orthotopic FUCCI Imaging Unveils the Adverse Effect of Irradiation Therapy

Radiotherapy is an important part of breast cancer treatment. Radiation therapy is well known to kill cancer cells, sometimes eradicate tumors, and then reduce the recurrence rate and prolong overall survival. However, radiation is recognized to induce chronic inflammation, which increases the risk of developing several types of cancer, including breast cancer. Bouchard et al. [71] reported that pre-irradiation of the mammary gland of nude mice promoted the migration of D2A1 FUCCI-expressing breast cancer cells using intravital imaging. Intravital FUCCI imaging showed that pre-irradiation promotes the migration of FUCCI-red cancer cells while reducing the FUCCI-green proliferative cancer cells. These results suggested that FUCCI-red quiescent cancer cells are resistant to irradiation and play a key role in metastasis. Furthermore, Bouchard et al. showed that pre-irradiation of mammary gland promotes lung metastasis [71]. Onozato et al. [72] demonstrated using FUCCI real-time imaging of tumor spheroids that FUCCI-green proliferative cancer cells located at the outside of the spheroids are sensitive to irradiation; in contrast, FUCCI-red cancer cells located in the center of the spheroids are dormant 40 days after irradiation, and then survive for more than two months, indicating that they are radioresistant. 

## 6. Intravital Orthotopic FUCCI Imaging Identifies Cell Cycle-Related Genes

Kagawa et al. [73] demonstrated with intravital multiphoton microscopy and FUCCI imaging cell cycle-associated cancer cell mobilization and invasion. S/G_2_/M-phase FUCCI-green proliferating cells, but not G_0_/G_1_-phase FUCCI-red quiescent cells, invaded surrounding tissues. Kagawa et al. also performed cDNA microarray-based comparative analyses between FUCCI-green and -red cells in culture and in vivo to elucidate the molecular mechanism of the control of cell cycle-dependent motility. Arhgap11A was identified as a cell cycle-dependent mobility-controlling molecule [73]. 

## 7. New Cell-Cycle-Based Approaches to Treatment of Solid Tumor: Decoy, Trap, and Shoot Therapy

**Figure 6:**
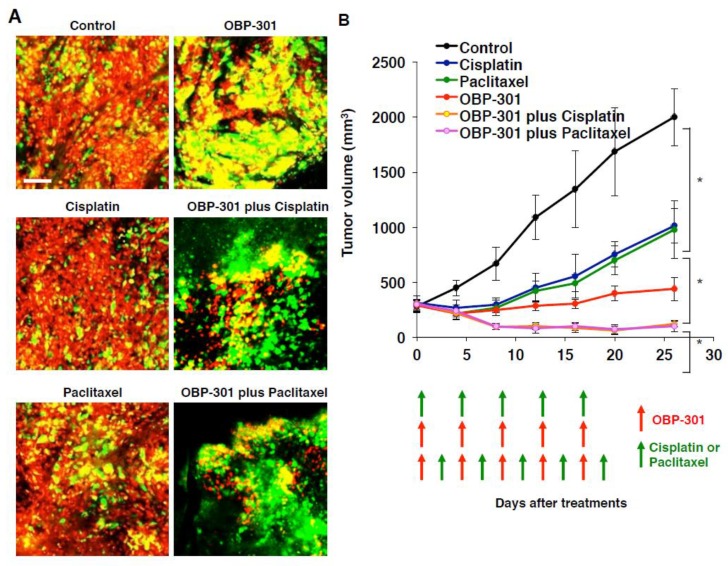
Oncolytic adenovirus, OBP-301, decoys quiescent cancer stem cells to cycle and become chemosensitive. FUCCI-expressing MKN45 cells (5 × 10^6^ cells/mouse) were injected subcutaneously into the left flank of mice. When the tumors reached approximately 8 mm in diameter (tumor volume, 300 mm^3^), mice were administered OBP-301 intratumorally (1 × 10^8^ PFU/tumor) and injected intraperitoneally with cisplatinum (CDDP) (4 mg/kg) or paclitaxel (PTX) (5 mg/kg) for five cycles every 3 days. (**A**) Representative images of cross-sections of FUCCI-expressing MKN45 subcutaneous tumor of control, OBP-301-, CDDP-, PTX-, or the combination of OBP-301 and chemotherapy-treated mice. The cells in G_0_/ G_1_, S, or G_2_/M phases appear red, yellow, or green, respectively. (**B**) Tumor growth curves are derived from FUCCI-expressing MKN45 cells after treatment with chemotherapy, OBP-301, or the combination of OBP-301 and chemotherapy. Red and green arrows indicate the days of treatment with OBP-301 and chemotherapy, respectively. Data are shown as means ± SD (*n* = 6). * *p* < 0.05, ANOVA. Scale bars, 100 μm. (Reproduced from [74] with the permission of American Association Cancer Research).

**Figure 7:**
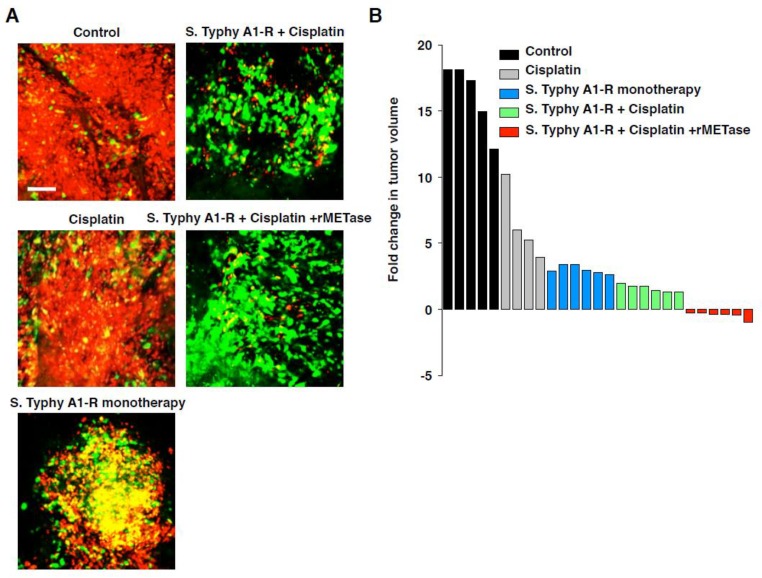
Decoy, trap, and shoot chemotherapy guided by FUCCI imaging. FUCCI-expressing MKN45 cells (5 × 10^6^ cells/mouse) were injected subcutaneously into the left flank of nude mice. When the tumors reached approximately 8 mm in diameter (tumor volume, 300 mm^3^), mice were administered *S. typhimurium* A1-R i.v. alone; or with CDDP (5 mg/kg i.p.) alone for 5 cycles every 7 days, or with a combination of *S. typhimurium* A1-R and CDDP; or with a combination of *S. typhimurium* A1-R, rMETase (200 U/day, for 3 days/cycle, 5 cycle), and CDDP (5 mg/kg, i.p.). (**A**) Representative images of cross-sections of FUCCI-expressing MKN45 tumors; untreated control; *S. typhimurium* A1-R-treated; CDDP-treated; or treated with the combination of *S. typhimurium* A1-R and CDDP; or the combination of *S. typhimurium* A1-R, rMETase, and CDDP. (**B**) Waterfall plot indicating fold change in tumor volume with each treatment. (Reproduced from [80] with the permission of Taylor and Francis).

Intravital FUCCI imaging demonstrated that quiescent, chemoresistant cancer cells should be targeted. Therefore, it was hypothesized that mobilizing the cancer-cell cycle from G_0_/G_1_ phase to S/G_2_/M phase would make the cancer cells sensitive to DNA damage drugs or to antimitotic drugs. Yano et al. [74] demonstrated that a telomerase-specific oncolytic adenovirus, OBP-301 [75,76], decoyed and trapped formerly quiescent cancer cells in early S phase and sensitized the decoyed cancer cells to currently-used cytotoxic anticancer agents (Figure 6). Yano et al. [77] also showed with FUCCI imaging that when cancer cells were treated with recombinant methioninase (rMETase), the cancer cells were selectively trapped in S/G_2_ (Figure 7). Moreover, Yano et al. [78] also showed that cancer-targeting *Salmonella typhinurium* A1-R [79] also decoyed cancer cells from G_0_/G_1_ phase to G_2_/M phase (Figure 7), and when cancer cells were subsequently treated with recombinant methioninase (rMETase), the cancer cells were selectively trapped in S/G_2_ (Figure 7) [80]. Tumors decoyed by *S. typhinurium* A1-R and trapped by rMETase were significantly more sensitive to conventional chemotherapy than cancer cells that were not pretreated with this strategy (Figure 7).

## 8. Conclusions

Intravital real-time monitoring of FUCCI-expressing tumors demonstrated cell-cycle dynamics of each cancer cell in a tumor in a live animal, suggesting why current cytotoxic agents are mostly ineffective. FUCCI imaging enabled us to develop a curative strategy to overcome cancer-cell quiescence in tumors.

## References

[1] Hoffman R.M. (2005). The multiple uses of fluorescent proteins to visualize cancer in vivo. Nat. Rev. Cancer.

[2] Hoffman R.M., Yang M. (2006). Color-coded fluorescence imaging of tumor host interactions. Nat Protoc..

[3] Newman R.H., Fosbrink M.D., Zhang J. (2011). Genetically encodable fluorescent biosensors for tracking signaling dynamics in living cells. Chem. Rev..

[4] Condeelis J., Segall J.E. (2003). Intravital imaging of cell movement in tumours. Nat. Rev. Cancer.

[5] Chishima T., Miyagi Y., Wang X., Yamaoka H., Shimada H., Moosa A.R., Hoffman R.M. (1997). Cancer invasion and micrometastasis visualized in live tissue by green fluorescent protein expression. Cancer Res..

[6] Chishima T., Miyagi Y., Wang X., Yamaoka H., Shimada H., Moosa A.R., Hoffman R.M. (1997). Visualization of the metastatic process by green fluorescent protein expression. Anticancer Res..

[7] Chishima T., Miyagi Y., Wang X., Yamaoka H., Shimada H., Moosa A.R., Hoffman R.M. (1997). Metastatic patterns of lung cancer visualized live and in process by green fluorescence protein expression. Clin. Exp. Metastasis.

[8] Yang M., Hasegawa S., Jiang P., Wang X., Tan Y., Chishima T., Shimada H., Moosa A.R., Hoffman R.M. (1998). Widespread skeletal metastatic potential of human lung cancer revealed by green fluorescent protein expression. Cancer Res..

[9] Yang M., Jiang P., Sun F.X., Hasegawa S., Baranov E., Chishima T., Shimada H., Moosa A.R., Hoffman R.M. (1999). A fluorescent orthotopic bone metastasis model of human prostate cancer. Cancer Res..

[10] Yang M., Jiang P., An Z., Baranov E., Li L., Hasegawa S., Chishima T., Shimada H., Moosa A.R., Hoffman R.M. (1999). Genetically fluorescent melanoma bone and organ metastasis models. Clin. Cancer Res..

[11] Yang M., Chishima T., Wang X., Baranov E., Shimada H., Moosa A.R., Hoffman R.M. (1999). Multi-organ metastatic capability of Chinese hamster ovary cells revealed by green fluorescent protein (GFP) expression. Clin. Exp. Metastasis.

[12] Bouvet M., Tsuji K., Yang M., Jiang P., Moosa A.R., Hoffman R.M. (2006). In vivo color-coded imaging of the interaction of colon cancer cells and splenocytes in the formation of liver metastasis. Cancer Res..

[13] Naumov G.N., Wilson S.M., MacDonald I.C., Schmidt E.E., Morris V.L., Groom A.C., Hoffman R.M., Chambers A.F. (1999). Cellular expression of green fluorescent protein, coupled with high-resolution in vivo videomicroscopy, to monitor steps in tumor metastasis. J. Cell Sci..

[14] Yang M., Baranov E., Moosa A.R., Penman S., Hoffman R.M. (2000). Visualizing gene expression by whole-body fluorescence imaging. Proc. Natl. Acad. Sci. USA.

[15] Yang M., Baranov E., Jiang P., Sun F.X., Li X.M., Li L., Hasegawa S., Chishima T., Shimada H., Moosa A.R. (2000). Whole-body optical imaging of green fluorescent protein-expressing tumors and metastasis. Proc. Natl. Acad. Sci. USA.

[16] Tsien R. (1998). The green fluorescence protein. Anuu. Rev. Biochem..

[17] Yamauchi K., Yang M., Jiang P., Yamamoto N., Xu M., Amoh Y., Tsuji K., Bouvet M., Tsuchiya H., Tomita K. (2006). Development of real-time subcellular dynamics multicolor imaging of cancer-cell tracking in live mice with a variable-magnification whole-mouse imaging system. Cancer Res..

[18] Yang M., Baranov E., Li X.M., Wang J.W., Jiang P., Li L., Moosa A.R., Penman S., Hoffman R.M. (2001). Whole-body and intravital optical imaging of angiogenesis in orthotopiccally implanted tumors. Proc. Natl. Acad. Sci. USA.

[19] Yang M., Baranov E., Wang J.W., Jiang P., Wang X., Sun F.X., Bouvet M., Moosa A.R., Penman S., Hoffman R.M. (2002). Direct external imaging of nascent cancer, tumor progression, angiogenesis, and metastasis on internal organs in the fluorescent orthotopic model. Proc. Natl. Acad. Sci. USA.

[20] Bouvet M., Wang J., Nardin S.R., Nassirpour R., Yang M., Baranov E., Jiang P., Moosa A.R., Hoffman R.M. (2002). Real-time optical imaging of primary tumor. Cancer Res..

[21] Yamamoto N., Jiang P., Yang M., Xu M., Yamauchi K., Tsuchiya H., Tomita K., Geoffrey M., Abdool R.W., Moosa A.R. (2004). Cellular dynamics visualized in live cells in vitro and in vivo by differential dual-color nuclear-cytoplasmic fluorescent-protein expression. Cancer Res..

[22] Yang M., Jiang P., Hoffman R.M. (2007). Whole-body subcellular multicolor imaging of tumor-host interaction and drug response in real time. Cancer Res..

[23] Yamamoto N., Yang M., Jiang P., Xu M., Tsuchiya H., Tomita K., Moosa A.R., Hoffman R.M. (2003). Determination of clonality of metastasis by cell-specific color-coded fluorescent-protein imaging. Cancer Res..

[24] Yang M., Reynoso J., Jiang P., Li L., Moosa A.R., Hoffman R.M. (2004). Transgenic nude mouse with ubiquitous green fluorescent protein expression as a host for human tumors. Cancer Res..

[25] Amoh Y., Yang M., Li L., Reynoso J., Bouvet M., Moossa A.R., Katsuoka K., Hoffman R.M. (2005). Nestin-linked green fluorescent protein transgenic nude mouse for imaging human tumor angiogenesis. Cancer Res..

[26] Schepers A.G., Snippert H.J., Stange D.E., van den Born M., van Es J.H., van de Wetering M., Clevers H. (2012). Lineage tracing reveals Lgr5+ stem cell activity in mouse intestinal adenomas. Science.

[27] Livet J., Weissman A., Kang H., Draft W., Lu J., Bennis R.A., Sanes J.R., Lichtman J.W. (2007). Transgenic strategies for combinatorial expression of fluorescent proteins in the nervous system. Nature.

[28] Timpson P., McGhee E.J., Anderson K. (2011). Imaging molecular dynamics in vivo-from cell biology to animal models. J. Cell Sci..

[29] Nobis M., Warren S.C., Luca M.C., Murphy K.J., Herrrman D., Timpson P. (2018). Molecular mobility and activity in an intravital imaging setting-implications for cancer progression and targeting. J. Cell Sci..

[30] Weissleder R., Pittet M.J. (2008). Imaging in the era of molecular oncology. Nature.

[31] Carragher N. (2011). Live cell in vitro and in vivo imaging applications: Accelerating drug discovery. Pharmaceutics.

[32] Kamb A. (2005). What’s wrong with our cancer models?. Nat. Rev. Drug Discov..

[33] Beerling E., Ritsma L., Vrisekoop N., Derksen P.W., van Rheenen J. (2011). Intravital microscopy: New insights into metastasis of tumors. J. Cell Sci..

[34] Barretto R.P., Ko T.H., Jung J.C., Wang T.J., Capps G., Waters A.C., Ziv Y., Attardo A., Recht L., Schnitzer M.J. (2011). Time-lapse imaging of disease progression in deep brain areas using fluorescence microendoscopy. Nat. Med..

[35] Kedrin D. (2008). Intravital imaging of metastatic behavior through a mammary imaging window. Nat. Methods.

[36] Sakaue-Sawano A., Kurokawa H., Morimura T., Tanyu A., Osawa H., Kashiwagi S., Fukami K., Miyata T., Miyoshi H., Imamura T. (2008). Visualizing spatiotemporal dynamics of multicellular cell-cycle progression. Cell.

[37] Tomura M., Sakaue-Sawano A., Mori Y., Takase-Utsuji M., Hata A., Ohtawa K., Kanagawa A., Miyawaki A. (2013). Contrasting quiescent G0 phase with mitotic cell cycling in the mouse immune system. PLoS ONE.

[38] Sakaue-Sawano A., Kobayashi T., Ohtawa K., Miyawaki A. (2011). Drug-induced cell cycle modulation leading to cell-cycle arrest, nuclear mis-segregation, or endoreplication. BMC Cell Biol..

[39] Bajar B.T., Lam A.J., Badiee R.K., Oh Y.H., Chu J., Zhou X.X., Kim N., Kim B.B., Chung M., Yablonovitch A.L. (2016). Fluorescent indicators for simultaneous reporting of all four cell cycle phases. Nat. Methods.

[40] Sakaue-Sawano A., Yo M., Komatsu N., Hiratsuka T., Kogure T., Hoshida T., Goshima N., Matsuda M., Miyoshi H., Miyawaki A. (2017). Genetically Encoded Tools for Optical Dissection of the Mammalian Cell Cycle. Mol. Cell.

[41] Oki T., Nishimura K., Kitaura J., Togami K., Maehara A., Izawa K. (2014). A novel cell-cycle-indicator, mVenus-p27K-, identifies quiescent cells and visualizes G0-G1 transition. Sci. Rep..

[42] Chittajallu D.R., Florian S., Kohler R., Iwamoto Y., Orth J.D., Weissleder R., Danuse G., Mitchison T.J. (2015). In vivo cell-cycle profiling in xenograft tumors by quantitative intravital microscopy. Nat. Methods.

[43] Ritsma L., Steller E.J.A., Beerling E., Loomans C., Zomer A., Gerlach C., Vriekoop N., Seinstra D., Van Gurp L., Schafer R. (2012). Intravital microscopy through an abdominal imaging window reveals a pre-micrometastasis stage during liver metastasis. Sci. Transl. Med..

[44] Ritsma L., Steller E.J.A., Ellenbroek S.I.J., Kranenburg O., Borel Rinkes I.H.M., Van Rheenen J. (2013). Surgical implantation of an abdominal imaging window for intravital microscopy. Nat. Protoc..

[45] Bochner F., Fellus-Alyagor L., Kalchenko V., Shinar S., Neeman M. (2015). A novel intravital imaging window for longitudinal microscopy of the mouse ovary. Sci. Rep..

[46] Yano S., Zhang Y., Miwa S., Tome Y., Hiroshima Y., Uehara F., Yamamoto M., Suetsugu A., Kishimoto H., Tazawa H. (2014). Spatial-temporal FUCCI imaging of each cell in a tumor demonstrates locational dependence of cell cycle dynamics and chemoresponsiveness. Cell Cycle.

[47] Marusyk A., Almendro V., Polyak K. (2012). Intra-tumour heterogeneity: A looking glass for cancer?. Nat. Rev. Cancer.

[48] Gonzalez-Garcia I., Sole R.V., Costa J. (2002). Metapopulation dynamics and spatial heterogeneity in cancer. Proc. Natl Acad. Sci..

[49] Jain R.K. (2005). Nomalization of Tumor Vasculature: An Emerging Concept in Antiangiogenic Therapy. Science.

[50] Kienast Y., von Baumgarten L., Fuhrmann M., Klinkert W.E., Goldbrunner R., Herms J., Winkler F. (2010). Real-time imaging reveals the single steps of brain metastasis formation. Nat. Med..

[51] Yano S., Takehara K., Tazawa H., Kishimoto H., Urata Y., Kagawa S., Fujiwara T., Hoffman R.M. (2017). Cell-cycle-dependent drug-resistant quiescent cancer cells induce tumor angiogenesis after chemotherapy as visualized by real-time FUCCI imaging. Cell Cycle.

[52] Watson J.D. (2011). Curing “incurable” cancer. Cancer Discov..

[53] Gottesman M.M. (2002). Mechanisms of cancer drug resistance. Annu. Rev. Med..

[54] Aguirre-Ghiso J.A. (2007). Models, mechanisms and clinical evidence for cancer dormancy. Nat. Rev. Cancer.

[55] Goss P.E., Chambers A.F. (2010). Does tumour dormancy offer a therapeutic target?. Nat. Rev. Cancer.

[56] Aguirre-Ghiso J.A., Bragado P., Sosa M.S. (2013). Metastasis Awakening: Targeting dormant cancer. Nat. Med..

[57] Polzer B., Klein C.A. (2013). The challenges of targeting minimal residual cancer. Nat. Med..

[58] Kreso A., O’Brien C.A., van Galen P., Gan O.I., Notta F., Brown A.M., Ng K., Ma J., Wienholds E., Dunant C. (2013). Viable clonal repopulation dynamics influence chemotherapy response in colorectal cancer. Science.

[59] Ishikawa F., Yoshida S., Saito Y., Hijikata A., Kitamura H., Tanaka S., Nakamura R., Tanaka T., Tomiyama H., Saito N. (2007). Chemotherapy-resistant human AML stem cells home to and engraft within the bone-marrow endosteal region. Nat. Biotechnol..

[60] Saito Y., Kitamura H., Hijikata A., Tomizawa-Murasawa M., Tanaka S., Takagi S., Uchida N., Suzuki N., Sone A., Najima Y. (2010). Identification of therapeutic targets for quiescent, chemotherapy-resistant human leukemia stem cells. Sci. Translational Med..

[61] Nakasone E.S., Askautrud H.A., Kees T., Park J.H., Plaks V., Ewald A.J., Fein M., Rasch M.G., Tan Y.X., Qiu J. (2012). Imaging tumor-stroma interaction during chemotherapy reveals contributions of the microenvironment to resistance. Cancer Cell.

[62] Giedt R. J., Koch P.D., Weissleder R. (2013). Single cell analysis of drug distribution by intravital imaging. PLoS ONE.

[63] Conway J.R.W., Carragher D.O., Timpson P. (2014). Developments in preclinical cancer imaging: Innovating the discovery of therapeutics. Nat. Rev. Cancer.

[64] Dan S., Okamura M., Mukai Y., Yoshimi H., Inoue Y., Hanyu A., Sakaue-Sawano A., Imamura T., Miyawaki A., Yamori T. (2012). ZSTK474, a specific phosphatidylinositol 3-kinase inhibitor, induces G1 arrest of the cell cycle in vivo. Eur. J. Cancer..

[65] Thurber G.M., Yang K.S., Reiner T., Kohler R.H., Sorger P., Mitchison T., Weissleder R. (2013). Single-cell and subcellular pharmacokinetic imaging allows insight into drug action in vivo. Nat. Commun..

[66] Bao Y., Mukai K., Hishiki T., Kuo A., Ohmura M., Sugiura Y., Matsuura T., Nagahata Y., Hayakawa N., Yamamoto T. (2013). Energy management by enhanced glycolysis in G1-phase in human colon cancer cells in vitro and in vivo. Mol. Cancer Res..

[67] Haass N.K., Beaumont K.A., Hill D.S., Anfosso A., Mrass P., Munoz M.A., Kinjo I., Weninger W. (2014). Real-time cell cycle imaging during melanoma growth, invasion, and drug response. Pigm. Cell Melanoma Res..

[68] Orth J.D., Kohler R.H., Foijer F., Sorger P.K., Weissleder R., Mitchison T.J. (2011). Analysis of Mitosis and Antimitotic Drug Responses in Tumors by In Vivo Microscopy and Single-Cell Pharmacodynamics. Cancer Res..

[69] Janssen A., Beerling E., Medema R., van Rheenen J. (2013). Intravital FRET imaging of tumor cell viability and mitosis during chemotherapy. PLoS ONE.

[70] Zasadil L.M., Andersen K.A., Yeum D., Rocque G.B., Wilke L.G., Tevaarwerk A.J., Burkard M.E., Weaver B.A. (2014). Cytotoxicity of paclitaxel in breast cancer is due to chromosome missegregation on multipolar spindles. Sci. Translational Med..

[71] Bouchard G., Bouvette G., Therriault H., Bujold R., Saucier C., Paquette B. (2013). Pre-irradiation of mouse mammary gland stimulates cancer cell migration and development of lung metastases. Br. J. Cancer..

[72] Onozato Y., Kaida A., Harada H., Miura M. (2017). Radiosensitivity of quiescent and proliferating cells grown as multicellular tumor spheroids. Cancer Sci..

[73] Kagawa Y., Matsumoto S., Kamioka Y., Mimori K., Naito Y., Ishii T., Okuzaki D., Nishida N., Maeda S., Naito A. (2013). Cell cycle-dependent Rho GTPase activity dynamically regulates cancer cell motility and invasion in vivo. PLoS ONE.

[74] Yano S., Tazawa H., Hashimoto Y., Shirakawa Y., Kuroda S., Nishizaki M., Kishimoto H., Uno F., Nagasaka T., Urata Y. (2013). A genetically engineered oncolytic adenovirus decoys and lethally traps quiescent cancer stem-like cells in S/G2/M phases. Clin. Cancer Res..

[75] Kawashima T., Kagawa S., Kobayashi N., Shirakiya Y., Umeoka T., Teraishi F., Taki M., Kyo S., Tanaka N., Fujiwara T. (2004). Telomerase-specific replication-selective virotherapy for human cancer. Clin. Cancer Res..

[76] Nemunaitis J., Tong A.W., Nemunaitis M., Senzer N., Phadke A.P., Bedell C., Adams N., Zhang Y.A., Maples P.B., Chen S. (2010). A phase I study of telomerase-specific replication competent oncolytic adenovirus (telomelysin) for various solid tumors. Mol. Ther..

[77] Yano S., Li S., Han Q., Tan Y., Bouvet M., Fujiwara T., Hoffman R.M. (2014). Selective methioninase-induced trap of cancer cells in S/G2 phase visualized by FUCCI imaging confers chemosensitivity. Oncotarget.

[78] Yano S., Zhang Y., Zhao M., Hiroshima Y., Miwa S., Uehara F., Kishimoto H., Tazawa H., Bouvet M., Fujiwara T. (2014). Tumor-targeting Salmonella typhimurium A1-R decoys quiescent cancer cells to cycle as visualized by FUCCI imaging and become sensitive to chemotherapy. Cell Cycle.

[79] Zhao M., Yang M., Li X.M., Jiang P., Baranov E., Li S., Xu M., Penman S., Hoffman R.M. (2005). Tumor-targeting bacterial therapy with amino acid auxotrophs of GFP-expressing Salmonella typhimurium. Proc. Natl. Acad. Sci. USA.

[80] Yano S., Takehara K., Zhao M., Tan Y., Han Q., Li S., Bouvet M., Fujiwara T., Hoffman R.M. (2016). Tumor-specific cell-cycle decoy by Salmonella typhimurium A1-R combined with tumor-selective cell-cycle trap by methioninase overcome tumor intrinsic chemoresistance as visualized by FUCCI imaging. Cell Cycle.

